# Cutaneous metastasis to the face from lung adenocarcinoma

**DOI:** 10.11604/pamj.2016.24.134.9504

**Published:** 2016-06-10

**Authors:** Sami Aziz Brahmi, Youssef Seddik

**Affiliations:** 1 Medical Oncology Service, Hospital Mohammed VI, Oujda, Morocco

**Keywords:** Cutaneous metastasis, face metastasis, lung cancer

## Image in medicine

Cutaneous metastases in the facial region occur in less than 0.5% of patients with metastatic cancer, and they usually originate from malignant melanoma. In this report, we describe an unusual case of lung adenocarcinoma metastasizing to his face at the time of initial diagnosis. The patient was 64-year-old man, a heavy smoker; he was referred to our department with a short history of dyspnea, pleuritic pain and loss of weight, as well as a nodule on his left cheek which was noticed simultaneously with the respiratory symptoms. His general condition was good. A chest X-ray revealed a left upper lobe mass with. Bronchoscopy with biopsy revealed a primitive adenocarcinoma of the lung. The patient underwent computed tomography (CT) scans of brain and abdomen, and chest. Radiological exams revealed a left upper lobe tumor with hepatic metastasis. The patient underwent excision biopsy of the facial lesion. Subsequent histological sections showed infiltration by lung adenocarcinoma confirmed by immunochemistry; TTF1 and CK7 positives. A palliative chemotherapy was initiated, the patient survived 6 months.

**Figure 1:**
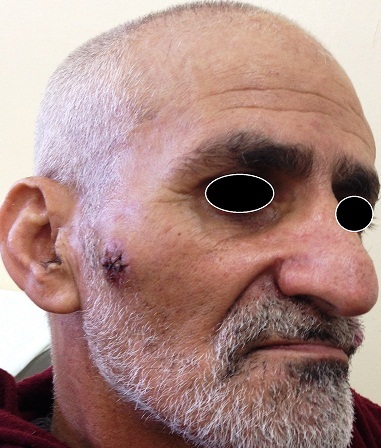
Image of facial cutaneous metastasis from lung adenocarcinoma

